# Edible Films Based on Fish Gelatin and Soluble Soybean Polysaccharide Enriched with Tea Polyphenol for Active Food Packaging

**DOI:** 10.3390/polym17162174

**Published:** 2025-08-08

**Authors:** Jie Liu, Zhongfeng Song, Yiwei Wang, Ying Pei, Keyong Tang

**Affiliations:** School of Materials Science and Engineering, Zhengzhou University, Zhengzhou 450001, China; songzhongfeng@gs.zzu.edu.cn (Z.S.); peiying@zzu.edu.cn (Y.P.)

**Keywords:** fish gelatin, soluble soybean polysaccharide, tea polyphenol, edible film, antioxidant activity

## Abstract

The increasing demand for environmentally friendly alternatives to conventional plastic packaging has driven interest in the development of biodegradable edible films with functional properties. In this work, edible blend films were developed based on fish gelatin (FG), soluble soybean polysaccharide (SSPS), and tea polyphenol (TP) for active food packaging applications. The FG/SSPS/TP films were prepared by solvent casting and characterized in terms of their structural, mechanical, optical, thermal, and barrier properties. FTIR, SEM, and XRD analyses revealed TP-induced morphological and structure changes in the biopolymer matrix. The incorporation of TP significantly enhanced the antioxidant activity and UV-shielding properties of the films, while also modifying their flexibility and surface hydrophilicity. The packaging performance of FG/SSPS/TP films was evaluated using beef tallow as a model food product. Compared to neat FG/SSPS and polyethylene films, the FG/SSPS/TP films effectively suppressed lipid oxidation of the samples during storage. The results demonstrated that the prepared FG/SSPS/TP films possess strong potential for use as edible and active packaging materials for food products.

## 1. Introduction

With the rapid development of ready-to-eat and convenience food products, biodegradable edible films have attracted increasing attention in recent years as sustainable alternatives to conventional synthetic plastic packaging [1]. These films are intended for direct consumption or safe food contact and are typically formulated from natural biopolymers, such as polysaccharides and proteins [2]. Compared to synthetic polymers, biopolymer-based films offer several advantages, including their broad availability from renewable resources, environmental friendliness, non-toxicity, and the potential to serve as carriers of functional additives, such as antioxidants and antimicrobials [3]. Their ability to act as a physical barrier against moisture, oxygen, and oil transfer makes them particularly suitable for food packaging applications. Moreover, increasing awareness of microplastic contamination has intensified public concern over food packaging safety [4], thereby enhancing the acceptance of, and even preference for, biopolymer-based edible films in direct food-contact applications.

Fish gelatin (FG) is a water-soluble protein derived from the partial hydrolysis of collagen in fish skin, scales or bones [5]. It has emerged as a promising alternative to mammalian gelatin for food-related applications in certain cultural and dietary contexts. Additionally, its production adds economic value to fish processing byproducts while contributing to waste reduction in the seafood industry [6]. Compared to mammalian gelatin, fish gelatin (FG) contains lower levels of proline and hydroxyproline, resulting in reduced thermal stability and gel strength [7]. Nevertheless, its excellent biodegradability, edibility, and compatibility with diverse functional additives render FG a promising protein for sustainable food packaging applications. FG is often combined with other biopolymers or functional ingredients to develop blend films with improved performance and reduced costs [8]. Various types of FG-based films have been developed by blending with materials such as chitosan [9], starch [10], alginate [11], and cellulose derivatives [12], or by incorporating bioactive compounds like plant extracts [13], essential oils [14], and polyphenols [15]. These composite systems have shown promising properties in extending shelf life, inhibiting microbial growth, and maintaining food quality, making FG a valuable biopolymer in the design of active and multifunctional packaging films.

Soluble soybean polysaccharide (SSPS), a water-soluble acidic polysaccharide extracted from okara (a residue generated during the production of soybean milk, tofu and other soybean products) [16], is considered as a readily accessible and cost-effective biopolymer derived from agricultural byproducts. SSPS is a structurally complex polysaccharide mainly composed of arabinose, galactose, and galacturonic acid. It features a compact, globular structure with a backbone consisting of long-chain rhamnogalacturonan and short-chain homogalacturonan regions [17,18]. SSPS exhibits excellent film-forming and adhesive properties, making it a promising material for the development of films for food and pharmaceutical applications [19,20,21,22]. However, neat SSPS films often exhibit inadequate mechanical strength, high water sensitivity, poor heat sealability, and limited functional activity, thereby limiting their practical applications [17,21]. We have demonstrated that blending porcine gelatin with SSPS is an effective approach to enhance the performance of SSPS-based edible films [23,24]. The SSPS/gelatin matrix exhibits favorable mechanical strength, water solubility, flexibility and heat sealability, all of which contribute to its suitability for food packaging applications.

In our previous study, we investigated the pH-induced complexation between FG and SSPS under acidic conditions, leading to the formation of coacervate-based edible films with improved structural and functional properties [19]. In this work, we aimed to develop edible films suitable for the packaging of lipid-rich food products by combining FG, SSPS, and tea polyphenols (TPs). TPs are natural compounds that are primarily extracted from green tea leaves and are widely used in combination with biopolymers such as gelatin [15], starch [25], and chitosan [26] to prepare biopolymer-based blend films with enhanced functional properties. Their chemical composition is dominated by catechins such as epigallocatechin gallate (EGCG), epicatechin (EC), and epicatechin gallate (ECG), which are responsible for their bioactivity and health-promoting effects [27]. In contrast to our previously reported coacervate-based FG/SSPS films, the present study focuses on the fabrication of FG/SSPS/TP composite films under neutral pH conditions in order to preserve the stability and antioxidant activity of TPs, which are prone to degradation in acidic environments. The prepared FG/SSPS/TP composite films were characterized in terms of their structural, mechanical, optical, thermal, barrier, and antioxidant properties. Furthermore, beef tallow packaging tests in heat-sealed pouches were performed to evaluate the practical applicability of the FG/SSPS/TP films. Overall, this work offers a novel strategy for developing clean-label, antioxidant-active packaging materials tailored to the needs of lipid-rich ready-to-eat and convenience foods.

## 2. Materials and Methods

### 2.1. Materials

Fish gelatin (FG) from cold-water fish skin (molecular weight: 60 kDa; CAS number: 9000-70-8; IEP: ~6.2; true density: ~1.35 g/cm^3^) in powder form was purchased from Sigma-Aldrich (St. Louis, MO, USA). Soluble soybean polysaccharide (molecular weight 410–650 kDa, residual protein content < 8%; true density: ~1.51 g/cm^3^) in powder form was kindly provided by JinJing Bio. Co., Ltd. (Pingdingshan, China). Tea polyphenol (99.0%, CAS number: 84650-60-2, green to yellow to brown powder) and glycerol were obtained from Macklin (Shanghai, China). 2,2′-Azino-bis (3-ethylbenzothiazoline-6-sulfonic acid) (ABTS) and 2,2-Diphenyl-1-picrylhydrazyl (DPPH) were obtained from Aladdin (Shanghai, China). All chemicals and reagents were used as received without additional treatment.

### 2.2. Film Preparation

The film preparation conditions were selected based on our previous work [19]. First, the FG was dissolved in 100 mL of deionized water at 40 °C using a thermostatic magnetic stirring water bath for 1 h to obtain a 3% (*w*/*v*) solution. Separately, SSPS was dissolved in 100 mL of deionized water at 80 °C for 2 h to prepare a 1% (*w*/*v*) solution. The two solutions were subsequently mixed under continuous stirring at 40 °C to achieve a homogeneous mixture with an FG-to-SSPS mass ratio of 3:1. TP was then added to the mixture of FG/SSPS at concentrations of 1%, 3%, or 5% (*w*/*w*, based on the total mass of biopolymers), followed by additional stirring for 10 min. The pH of the solution was adjusted to 7.0 using 1 mol/L NaOH solution. Glycerol (30% *w*/*w* based on the total mass of biopolymers) was added as a plasticizer to the mixture. The obtained film-forming solution was poured into a plastic Petri dish and dried at 30 °C for 24 h. The resulting films were carefully peeled off and stored for further use. For comparison, FG/SSPS film was prepared using the same procedure without the addition of TP. The film formulations are summarized in Table 1. The FG/SSPS/TP films containing 1%, 3% and 5% TP were coded as FG/SSPS/TP1, FG/SSPS/TP3 and FG/SSPS/TP5, respectively.

### 2.3. Film Characterization

FTIR spectra of the FG/SSPS-based films were collected using a Nicolet iS20 spectrometer (Thermo Fisher Scientific, Waltham, MA, USA) equipped with an attenuated total reflectance (ATR) accessory. Spectral data were acquired over the range of 4000–600 cm^−1^ with a resolution of 4 cm^−1^ and an accumulation of 32 scans.

X-ray diffraction (XRD) patterns were obtained using an EmpyreanX diffractometer (PANalytical B.V., Almelo, The Netherlands) equipped with a Cu Kα radiation source (λ = 1.54 Å) operated at 40 kV and 40 mA. Film samples were scanned from 5° to 90° (2θ) at a rate of 0.3°/s.

Surface and cross-sectional morphologies of the FG/SSPS-based films were observed using a Regulus 8100 scanning electron microscope (Hitachi, Tokyo, Japan) operated at an accelerating voltage of 10.0 kV. Film samples were cryo-fractured in liquid nitrogen to obtain fracture surfaces, then mounted on aluminum stubs using conductive adhesive and sputter-coated with a thin layer of gold prior to imaging.

The UV-Vis spectra of the films were recorded using a dual-beam UV–Vis spectrophotometer (TU-1901, Purkinje General Instrument Co., Ltd., Beijing, China) in the wavelength range of 200−850 nm. Films were cut into 30 mm × 15 mm rectangles and mounted on the sample holder, using air as the reference. CIELAB color parameters of the films, including L* (lightness), a* (red–green), and b* (yellow–blue), were measured using a portable digital colorimeter (JZ-500, Kingwell, Shenzhen, China) against a standard white background. The total color difference (ΔE) was calculated according to the following equation:

(1) $$∆E=\sqrt{{\left(L^{*}-L_{white}^{*}\right)}^{2}+{\left(a^{*}-a_{white}^{*}\right)}^{2}+{\left(b^{*}-b_{white}^{*}\right)}^{2}}$$

where the subscript *white* indicates the corresponding parameters of the standard white background.

Film thickness was determined using a digital micrometer with a precision of 0.001 mm. Measurements were performed at ten different points on each film in triplicate, and the mean value was calculated.

Mechanical properties of the FG/SSPS-based films, including tensile strength (TS), elongation at break (EB), and Young’s modulus (YM), were determined using a texture analyzer (SMSTA.XTPLUS, Stable Micro Systems, Godalming, UK) in tensile mode at a crosshead speed of 10 mm/min. Dumbbell-shaped specimens (gauge length: 30 mm, width: 4 mm) were conditioned at 58 ± 2% relative humidity (RH) and room temperature (RT) for 7 days prior to testing. Each measurement was performed in five replicates.

The moisture uptake capacity of the FG/SSPS-based films at RT was evaluated by determining water content (WC) following a previously established method [21]. For water solubility (WS) determination, pre-dried film specimens were immersed in 20 mL of deionized water at RT for 24 h. The undissolved film residues were then collected and dried at 105 ± 1 °C until a constant weight was reached. WS was calculated by comparing the initial dry weight (*W*_0_) with the final dry weight (*W_f_*) using the following equation:

(2) $$WS\left(\%\right)=\frac{W_{0}-W_{f}}{W_{0}}\times 100$$

The surface wettability of the FG/SSPS-based films was evaluated by measuring their static water contact angle (WCA) using a contact angle goniometer (model SPCAX2, HARKE Experimental Instrument Factory, Beijing, China). A droplet of deionized water (3.0 μL) was gently deposited onto the film surface, and the contact angle was recorded immediately to minimize the influence of evaporation and absorption. Each film sample was tested at 7 randomly selected positions, and the average WCA was calculated to ensure reliability and representativeness of the results.

Water vapor permeability (WVP) of the films was determined using a commercial permeability analyzer (W3/062, Labthink, Jinan, China). Film samples were mounted onto standard permeation cells containing 50 mL of deionized water and tightly sealed to allow only vertical vapor diffusion. The system automatically monitored mass changes of the cells over an 8 h period to determine water vapor transmission rate (WVTR), which was calculated from the linear portion of the mass loss versus time curve. WVP was then obtained using the following equation:

(3) $$WVP=\frac{WVTR\times L}{∆P\times A}$$

Thermogravimetric analysis (TGA) was performed to investigate the thermal properties of the films. Each film was cut into small pieces (≤1 mm in size), and approximately 5−10 mg was placed in an open aluminum crucible. The measurements were carried out under a nitrogen atmosphere (flow rate: 40 mL/min) using a heating program from 25 °C to 600 °C at a heating rate of 20 °C/min.

To evaluate the antioxidant activity of the FG/SSPS films, 0.1 g of film sample was immersed in 10 mL of deionized water for 24 h at RT to obtain the film extract. For the ABTS assay, the diluted film extract was mixed with ABTS solution at a volume ratio of 1:3 (*v*/*v*) and incubated in the dark for 1 h. The absorbance of the reaction mixture (*A*_1_) was then measured at 734 nm using a UV–Vis spectrophotometer. A control was prepared by mixing deionized water with ABTS at the same ratio and under identical conditions, and its absorbance was recorded as *A*_0_. For the DPPH assay, the film extract was mixed with DPPH solution at a volume ratio of 1:2 (*v*/*v*) and allowed to react in the dark for 30 min. The absorbance of the reaction mixture was measured at 517 nm. A similar procedure was followed for the control group using deionized water. The free radical scavenging activity was calculated using the following equation:

(4) $$Free\;radical\;scavenging\;activity\;(\%)=\frac{A_{0}-A_{1}}{A_{0}}\times 100$$

### 2.4. Packaging Application

The packaging applicability of the FG/SSPS/TP films was assessed using beef tallow as a model food product. The films were heat-sealed into semi-finished pouches (50 mm × 50 mm) using a laboratory-scale heat-sealing machine (HST-H6, Labthink Instruments Co. Ltd., China, Jinan, China) according to the method mentioned in our previous work [23]. Fresh beef tallow (5.0 g) was placed into semi-finished pouches and immediately heat-sealed to ensure airtight packaging. The sealed samples were stored at 35 ± 2 °C for 14 days. During storage, the peroxide value (POV) of beef tallow was determined at designated intervals according to the GB 5009.227–2016 standard [28] to monitor lipid oxidation. In this method, lipid peroxides react with potassium iodide to release iodine, which is subsequently quantified by titration with sodium thiosulfate.

### 2.5. Statistical Analysis

Data are presented as mean ± standard deviation and were analyzed by one-way analysis of variance (ANOVA) using SPSS 21 software (Chicago, IL, USA). Differences among groups were evaluated using Duncan’s multiple range test at a 95% confidence level (*p* < 0.05). All measurements were conducted in triplicate unless stated otherwise.

## 3. Results and Discussion

### 3.1. FTIR and XRD Analyses

**Figure 1:**
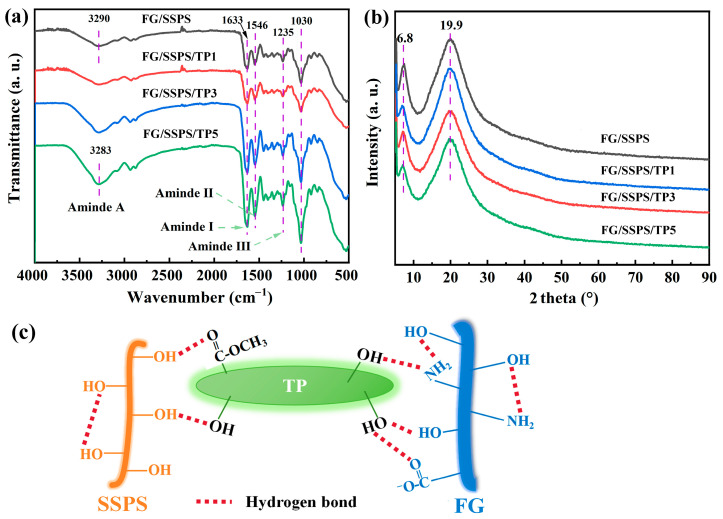
FTIR spectra (**a**) and XRD patterns (**b**) of FG/SSPS and FG/SSPS/TP films; schematic illustration of the hydrogen bonding interaction between TP and FG/SSPS molecules (**c**).

Figure 1a shows the FTIR spectra of FG/SSPS-based films. All films exhibited characteristic absorption bands corresponding to the protein and polysaccharide components. The broad peak around 3290 cm^−1^ is attributed to O-H and N-H stretching vibrations. The bands at 1633 cm^−1^, 1546 cm^−1^, and 1235 cm^−1^ correspond to amide I (C=O stretching), amide II (N-H bending and C-N stretching), and amide III (C-N stretching and N-H bending), respectively, confirming the incorporation of fish gelatin in the film matrix [29]. Additionally, the absorption peak at 1030 cm^−1^ is mainly associated with C-O-C stretching from the SSPS backbone [30]. With increasing TP content, the amide A band became broader and shifted slightly to lower wavenumbers, which may be attributed to enhanced hydrogen bonding interactions between TP and the FG/SSPS matrix [31]. These spectral changes confirm the successful incorporation of TP into the biopolymer matrix and suggest the occurrence of intermolecular interactions between TP and the biopolymer components. The potential hydrogen bonding interactions between TP and FG/SSPS matrix are illustrated in Figure 1c.

The XRD patterns of the FG/SSPS-based films in Figure 1b show two diffraction peaks centered at approximately 2θ = 6.8° and 19.9°. The broad peak centered at 2θ = 19.9° indicates that the FG/SSPS matrix exhibits a predominantly amorphous structure, while the peak at 2θ = 6.8° is mainly attributed to the triple-helix structure of gelatin formed during the drying of film-forming solution at relatively low temperature [32]. The incorporation of TP led to a decrease in the relative intensity ratio of the peak at 2θ = 6.8° to that at 19.9° (I_6.8°_/I_19.9°_), from 0.88 in the FG/SSPS film to 0.76 in the FG/SSPS/TP5 film, suggesting disruption of molecular packing and a reduction in the overall structural order. This effect is likely caused by the interference of TP, which disrupts the folding of gelatin chains into triple helices and facilitate the formation of a less ordered biopolymer network. Additionally, although glycerol is known to disrupt intermolecular interactions and reduce structural order due to its plasticizing effect, its content was kept constant across all film formulations. Therefore, the observed reduction in XRD peak intensity at 2θ = 6.8° is more likely attributed to the incorporation of TP rather than the influence of glycerol.

### 3.2. SEM Analysis

**Figure 2:**
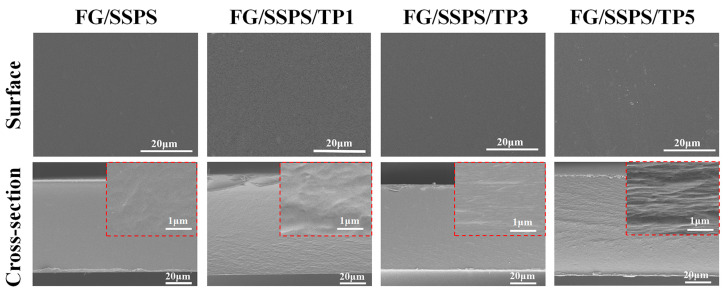
SEM micrographs of FG/SSPS and FG/SSPS/TP films; insets show cross-sectional images at higher magnification.

The surface and cross-sectional morphologies of the FG/SSPS-based films are presented in Figure 2. All films exhibited relatively smooth and homogeneous surfaces without visible cracks or pores, suggesting good compatibility between SSPS and fish gelatin. With the incorporation of TP, no significant changes in surface morphology were observed at low TP contents (1% and 3%). However, at a higher TP content (5%), the surface became slightly rougher with localized aggregation, which may be attributed to hydrogen bonding, hydrophobic interactions, and potential π–π stacking between TP and either FG or SSPS. These intermolecular interactions could promote partial phase separation or the formation of TP-rich domains within the film matrix. Cross-sectional micrographs revealed a dense and compact structure for the FG/SSPS-based film, whereas the addition of TP, particularly at higher levels, led to increased structural heterogeneity and the appearance of wrinkled regions, indicating that TP may disrupt molecular packing and weaken the internal cohesion of the biopolymer film. Similar findings were reported by Du et al. in their study on the effects of TP on the structural and physicochemical properties of rice starch [33]. These morphological changes are consistent with the XRD results mentioned above, supporting the hypothesis that TP incorporation reduces structural order and promotes the formation of a more amorphous and disordered network.

### 3.3. Optical Properties

**Figure 3:**
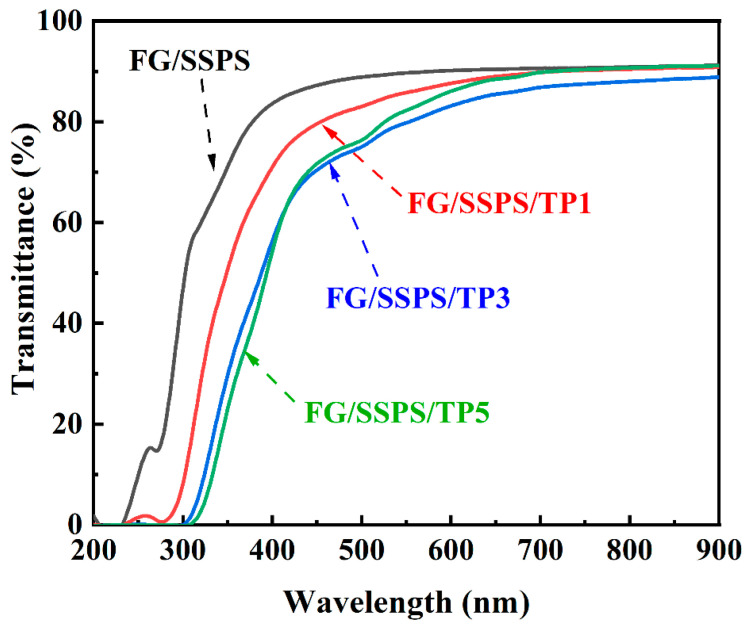
UV-Vis spectra of FG/SSPS and FG/SSPS/TP films.

Figure 3 shows the UV-Vis spectra of FG/SSPS and FG/SSPS/TP films. The neat FG/SSPS film exhibited the highest overall transmittance in the wavelength range of 200–900 nm, indicating excellent transparency. However, its UV-shielding ability was limited, with only low transmittance observed in the 200–250 nm region. After incorporation of TP, the UV-shielding performance of the FG/SSPS films improved significantly. All the films enriched with TP exhibited a marked decrease in transmittance in the wavelength range of 200–300 nm, with the FG/SSPS/TP3 and FG/SSPS/TP5 films showing nearly complete UV absorption in this region. The transmittance at 280 nm decreased from 19.7% in the TP-free film to 0.75%, 0.02%, and 0.01% in the FG/SSPS/TP1, FG/SSPS/TP3, and FG/SSPS/TP5 films, respectively, confirming the enhanced UV-shielding effect with increasing TP content. Despite the significantly enhanced UV-shielding properties, the FG/SSPS/TP films retained high transmittance in the visible spectrum (400–800 nm). Notably, even the film with the highest TP loading (FG/SSPS/TP5) exhibited a transmittance > 80% at 600 nm, demonstrating that effective UV-shielding was achieved without compromising optical clarity. This optimal balance between UV protection and visible-light transmittance makes these films suitable for food packaging applications, particularly for lipid-rich food products (e.g., dairy, meats, and edible oils), where UV-induced oxidation is one of the key factors contributing to spoilage [34].

**Figure 4:**
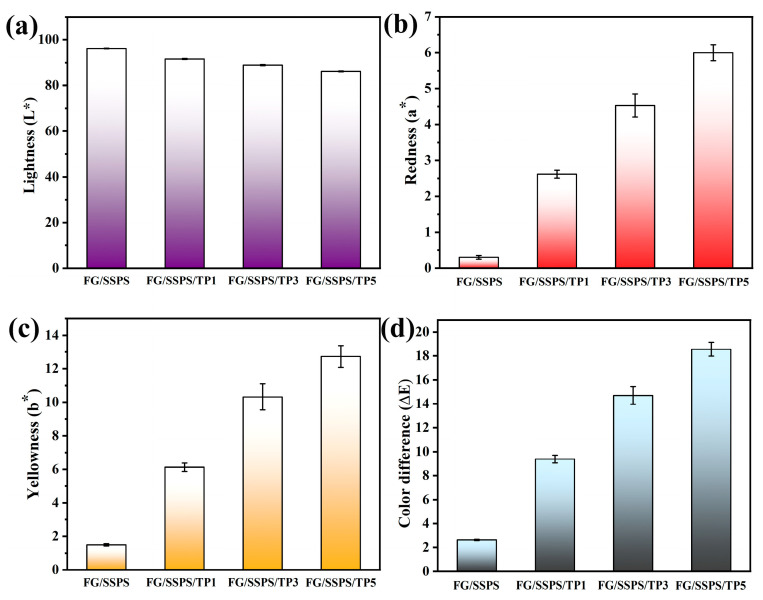
CIELAB color parameters of FG/SSPS and FG/SSPS/TP films.: L* (**a**), a* (**b**), b* (**c**), and ΔE (**d**).

Figure 4 shows the CIELAB color parameters (L*, a*, b*, and ΔE) of the FG/SSPS-based films. The L* value (lightness) slightly decreased with increasing TP content, indicating a minor darkening of the films. Meanwhile, the a* value (redness) and b* value (yellowness) both increased significantly with increasing TP content, indicating a noticeable color shift from a nearly colorless film (FG/SSPS) to a reddish-yellow hue in TP-containing films. This color change can be attributed to the natural pigmentation of TP, which mainly arises from oxidized products of catechins, such as theaflavins and thearubigins [35]. Correspondingly, the total color difference (ΔE) increased with higher TP concentrations, reaching its maximum in the FG/SSPS/TP5 film. These results indicate that while TP enhances the UV-shielding property of the FG/SSPS films, it also induces notable changes in optical appearance.

### 3.4. Thickness and Mechanical Properties

As shown in Table 2, there were no significant differences in the thickness of the films (*p* > 0.05), suggesting that the addition of 1–5% TP did not affect the structural compactness of the FG/SSPS films. The TS slightly but significantly decreased (*p* < 0.05) from 33.11 ± 1.53 MPa (FG/SSPS) to 26.16 ± 1.43 MPa with the addition of 5% TP, indicating a weakening of the biopolymer network. This reduction can be attributed to disruption of original hydrogen bonding and interference with the molecular packing between FG and SSPS caused by TP, as evidenced by the more disordered structure observed in the XRD patterns and the layered or rougher morphology revealed by SEM analysis. Although FTIR results indicate the formation of new hydrogen bonds between TP and biopolymers, these localized interactions are insufficient to fully compensate for the loss of the original, more extensive hydrogen-bonding network. In contrast, the EB increased significantly with the addition of TP, suggesting enhanced flexibility due to a less compact biopolymer network [36]. A similar trend was observed for YM, which decreased at low TP content but partially recovered at higher levels, possibly due to TP aggregation or phase separation that reinforced localized domains within the matrix. Collectively, the mechanical property measurements correlate well with the observed structural changes, indicating that TP induces reorganization of the film matrix architecture. This transformation reduces stiffness while enhancing deformability.

### 3.5. Water Content and Water Solubility

The WC and WS values of the FG/SSPS-based films are summarized in Table 3. The WC values ranged from 6.01% to 7.51%, with no statistically significant differences observed among the film samples (*p* > 0.05), indicating that TP incorporation had minimal effect on the moisture retention capacity of the films. Similarly, the addition of 1% and 3% TP had no significant influence on WS, suggesting that moderate TP levels did not markedly affect the water resistance of the film matrix. However, when TP content increased to 5%, a slight but statistically significant increase in WS was observed (*p* < 0.05). This increase may be attributed to structural loosening or phase separation at higher TP content, which could promote water diffusion and facilitate partial disintegration of the film in aqueous environments. These findings suggest that appropriate TP contents help maintain the water stability of FG/SSPS films, whereas excessive loading may compromise their structural integrity.

### 3.6. Water Contact Angle and Water Vapor Permeability

The WCA results provide insight into the surface wettability of the films. As shown in Table 3, the neat FG/SSPS film exhibited the highest WCA value (108.7 ± 4.2°), indicating a relatively hydrophobic surface. This hydrophobic characteristic has been commonly observed on gelatin-based films due to the orientation of hydrophobic groups or non-polar segments of gelatin molecules toward the air interface during the film-forming and drying process, resulting in a surface with reduced affinity for water [37]. Upon TP incorporation, the WCA values gradually decreased, with the FG/SSPS/TP5 film exhibiting the lowest value (61.7 ± 5.6°), indicating an enhancement in the surface hydrophilicity of the FG/SSPS matrix. This could be primarily attributed to the abundance of phenolic hydroxyl groups in TP, which may migrate toward the film surface during drying, enhancing surface polarity and thereby reducing the WCA. Additionally, the structural changes induced by TP incorporation, such as the more heterogeneous and layered morphology observed in SEM, may facilitate the rearrangement of biopolymer chains and promote the exposure of polar groups (particularly phenolic hydroxyl groups) on the film surface. This structural reorganization is also likely to contribute to the enhanced surface hydrophilicity and the reduced water contact angle observed in the FG/SSPS/TP films. Table 3 also shows the WVP of the FG/SSPS films before and after addition of TP. No significant differences were observed among the samples, indicating that the addition of TP had a negligible impact on the water vapor barrier properties of the FG/SSPS films.

### 3.7. Thermal Properties

**Figure 5:**
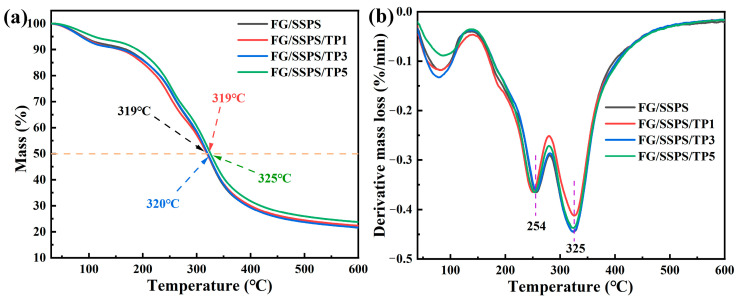
TG (**a**) and DTG (**b**) curves of FG/SSPS and FG/SSPS/TP films.

Figure 5 shows the thermogravimetry/derivative thermogravimetry (TG/DTG) curves of FG/SSPS-based films. All films exhibited a similar three-stage pattern during heating from room temperature (RT) to 600 °C. The first stage, occurring in the temperature range of RT-150 °C, corresponds to the evaporation of residual moisture and low-molecular-weight volatiles. The second stage (150–290 °C) is mainly attributed to the release of glycerol (plasticizer of the films) and represented by a weak DTG peak centered at around 254 °C. This assignment is consistent with the boiling point of glycerol (~290 °C) [38]. The third and major mass loss stage occurred between 290 °C and 500 °C, with a DTG maximum at around 325 °C for all films, corresponding to the thermal degradation of the FG/SSPS matrix and TP. The incorporation of TP did not significantly affect the characteristic degradation temperature, as evidenced by the nearly identical DTG peak temperatures. However, a slight improvement in thermal stability was observed in the FG/SSPS/TP5 film, which exhibited a higher temperature at 50% mass loss (325 °C) compared to other FG/SSPS-based films. This improvement in thermal stability could be attributed to the stabilizing effect of TP, likely resulting from hydrogen bonding interactions with the biopolymer matrix, which may restrict molecular mobility and thereby suppress thermal degradation. It is worth noting that this stabilizing effect was only evident at a TP content of 5%, whereas lower TP contents primarily led to increased flexibility, as indicated by the EB results, due to partial disruption of the original polymer network.

### 3.8. Antioxidant Activity

The antioxidant activity of FG/SSPS-based films was assessed using ABTS and DPPH radical scavenging assays, as shown in Table 4. The neat FG/SSPS film exhibited minimal radical scavenging capacity in both assays, indicating its limited intrinsic antioxidant properties. The incorporation of TP significantly enhanced antioxidant activity of the FG/SSPS-based films, with both ABTS and DPPH radical scavenging abilities increasing progressively with TP content. Specifically, the FG/SSPS/TP5 film exhibited the highest free radical scavenging activity, reaching 90.9 ± 2.6% in the ABTS assay and 67.4 ± 4.8% in the DPPH assay. This enhancement is mainly attributed to the abundant phenolic hydroxyl groups present in TP, which can donate hydrogen atoms or electrons to neutralize free radicals, thereby interrupting the propagation of oxidative chain reactions [39]. These findings confirm that the incorporation of TP effectively imparts dose-dependent antioxidant activity to the FG/SSPS-based films.

### 3.9. Packaging Application in Beef Tallow Preservation

**Figure 6:**
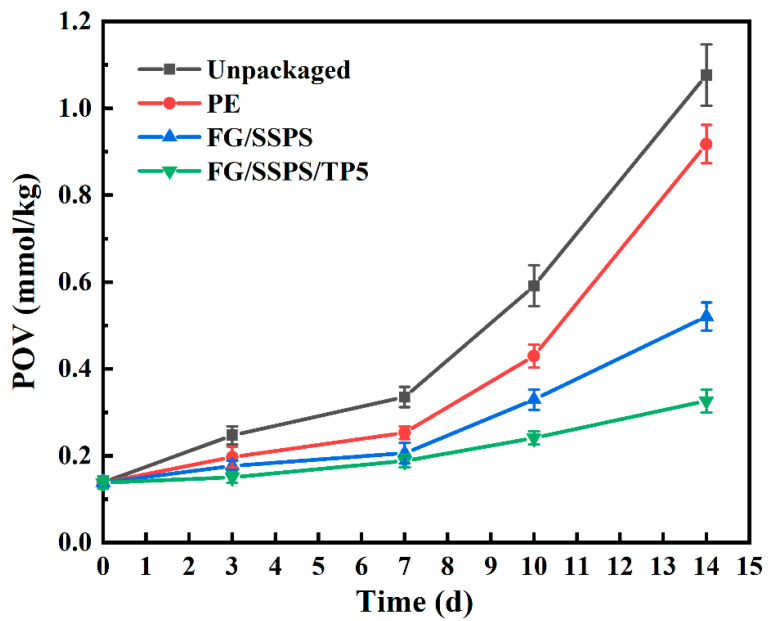
POV of beef tallow samples packaged with different films during 14 days of storage at 35 ± 2 °C.

The effectiveness of FG/SSPS/TP films in inhibiting lipid oxidation was evaluated by monitoring the peroxide value (POV) of beef tallow samples during 14 days of storage at 35 ± 2 °C (Figure 6). Beef tallow was selected due to its high lipid content, susceptibility to oxidation, and widespread use in convenience foods such as instant noodles and ready-to-eat meals, making it a representative food model for evaluating the packaging performance of the developed films. As expected, the POV of the unpackaged sample increased rapidly, reaching 1.1 mmol/kg by day 14, indicating evident oxidative deterioration. The beef tallow sample wrapped in commercial polyethylene (PE) film showed a moderate increase in POV, while the beef tallow packaged with neat FG/SSPS film exhibited improved oxidative stability. This finding is consistent with previous findings on gelatin-based packaging films [40]. Among all tested films, the FG/SSPS/TP5 film exhibited the most effective protection against lipid oxidation, consistently maintaining POV values below 0.35 mmol/kg throughout the entire storage period. Statistical analysis revealed significant differences (*p* < 0.05) between the FG/SSPS/TP5 and the other film groups, confirming its enhanced oxidative stability. This superior antioxidant performance is attributed to the presence of TP, which function as effective radical scavengers and hydrogen donors. The results demonstrate the strong potential of FG/SSPS/TP films as active food packaging materials. For instance, these edible films have potential applications in the packaging of lipid-containing seasonings commonly used in instant noodle products. In such cases, the films can be directly used without removal and consumed together with the noodles during soaking or cooking, providing both convenience and functional protection.

## 4. Conclusions

In this study, an edible composite film was successfully developed by incorporating tea polyphenols into fish gelatin and soluble soybean polysaccharide matrices. The resulting FG/SSPS/TP composite films exhibited enhanced antioxidant activity, favorable mechanical properties and improved UV-shielding performance without compromising optical transparency. Structural and morphological analyses revealed that TP induced a more amorphous and disordered film network, contributing to changes in mechanical properties and surface wettability. Notably, the TP-enriched FG/SSPS films demonstrated dose-dependent radical scavenging capacity and effectively delayed lipid oxidation in beef tallow packaging tests. These results highlight the potential of FG/SSPS/TP films as active and edible packaging materials, particularly suitable for lipid-rich or oxidation-sensitive food products. In summary, this work provides a sustainable strategy for developing functional biopolymer composite films with both protective and preservative functionalities.

## Figures and Tables

**Table 1:** Summary of film formulations *.

Films	FG (wt%)	SSPS (wt%)	TP (wt%)	Glycerol (wt%)
FG/SSPS	75.0	25.0	0.0	30
FG/SSPS/TP1			1.0	
FG/SSPS/TP3			3.0	
FG/SSPS/TP5			5.0	

* FG and SSPS were combined and normalized to 100 wt%; TP and glycerol were added at weight percentages relative to the total mass of FG and SSPS.

**Table 2:** Thickness, tensile strength (TS), elongation at break (EB), and Young’s modulus (YM) of FG/SSPS and FG/SSPS/TP films *.

Films	Thickness (μm)	TS (MPa)	EB (%)	YM (MPa)
FG/SSPS	85 ± 3 ^a^	33.11 ± 1.53 ^a^	11.00 ± 2.29 ^b^	868.26 ± 58.90 ^a^
FG/SSPS/TP1	89 ± 4 ^a^	28.44 ± 1.87 ^b^	21.17 ± 1.39 ^a^	676.64 ± 74.28 ^b^
FG/SSPS/TP3	85 ± 4 ^a^	28.40 ± 1.91 ^b^	19.76 ± 0.50 ^a^	683.75 ± 52.33 ^b^
FG/SSPS/TP5	89 ± 3 ^a^	26.16 ± 1.43 ^b^	20.72 ± 0.74 ^a^	866.08 ± 35.60 ^a^

* Different lowercase superscript letters in the same column indicate significant differences (*p* < 0.05).

**Table 3:** Water content (WC), water solubility (WS), water contact angle (WCA), and water vapor permeability (WVP) of FG/SSPS and FG/SSPS/TP films *.

Films	WC (%)	WS (%)	WCA (°)	WVP × 10^−10^ (g·m^−1^·s^−1^·Pa^−1^)
FG/SSPS	6.26 ± 1.40 ^a^	31.52 ± 1.17 ^b^	108.7 ± 4.2 ^a^	2.47 ± 0.12 ^a^
FG/SSPS/TP1	7.51 ± 0.47 ^a^	29.83 ± 0.63 ^b^	107.7 ± 3.7 ^a^	2.53 ± 0.09 ^a^
FG/SSPS/TP3	6.76 ± 0.70 ^a^	30.86 ± 0.71 ^b^	64.9 ± 1.9 ^b^	2.37 ± 0.06 ^a^
FG/SSPS/TP5	6.01 ± 0.18 ^a^	33.31 ± 0.89 ^a^	61.7 ± 5.6 ^b^	2.43 ± 0.05 ^a^

* Different lowercase superscript letters in the same column indicate significant differences (*p* < 0.05).

**Table 4:** ABTS and DPPH free radical scavenging activity of FG/SSPS and FG/SSPS/TP films *.

Films	ABTS (%)	DPPH (%)
FG/SSPS	7.4 ± 2.2 ^a^	1.1 ± 0.3 ^b^
FG/SSPS/TP1	23.3 ± 2.5 ^a^	16.9 ± 3.5 ^b^
FG/SSPS/TP3	70.3 ± 5.1 ^a^	50.8 ± 5.2 ^b^
FG/SSPS/TP5	90.9 ± 2.6 ^a^	67.4 ± 4.8 ^a^

* Different lowercase superscript letters in the same column indicate significant differences (*p* < 0.05).

## Data Availability

All data supporting the findings of this study are included in the article.
